# Harnessing IGF-1 and IL-2 as biomarkers for calcineurin activity to tailor optimal FK506 dosage in α-synucleinopathies

**DOI:** 10.3389/fmolb.2023.1292555

**Published:** 2023-11-29

**Authors:** Sofia Zaichick, Gabriela Caraveo

**Affiliations:** Department of Neurology, Feinberg School of Medicine, Northwestern University, Chicago, IL, United States

**Keywords:** α-synuclein, calcineurin, FK506, neuroprotection, biomarkers, IGF-1, IL-2

## Abstract

**Introduction:** Rise in Calcium (Ca^2+^) and hyperactive Ca^2+^-dependent phosphatase calcineurin represent two key determinants of a-synuclein (a-syn) pathobiology implicated in Parkinson’s Disease (PD) and other neurodegenerative diseases. Calcineurin activity can be inhibited with FK506, a Food and Drug Administration (FDA)-approved compound. Our previous work demonstrated a protective effect of low doses of FK506 against a-syn pathology in various models of a-syn related pathobiology.

**Methods:** Control and a-syn-expressing mice (12-18 months old) were injected with vehicle or two single doses of FK506 administered 4 days apart. Cerebral cortex and serum from these mice were collected and assayed using a meso scale discovery quickplex SQ 120 for cytokines and Enzyme-linked immunosorbent assay for IGF-1.

**Results**: In this study we present evidence that reducing calcineurin activity with FK506 in a-syn transgenic mice increased insulin growth factor (IGF-1), while simultaneously decreasing IL-2 levels in both cerebral cortex and serum.

**Discussion:** The highly conserved Ca^2+^/calcineurin signaling pathway is known to be affected in a-syn-dependent human disease. FK506, an already approved drug for other uses, exhibits high brain penetrance and a proven safety profile. IL-2 and IGF-1 are produced throughout life and can be measured using standard clinical methods. Our findings provide two potential biomarkers that could guide a clinical trial of FK506 in PD patients, without posing significant logistical or regulatory challenges.

## Introduction

Misfolding of the small lipid-binding protein, α-synuclein (α-syn), plays a central role in a group of neurological diseases collectively known as synucleinopathies (Alafuzoff and Hartikainen, 2017). These encompass Parkinson’s disease (PD), multiple system atrophy, and two prominent Lewy body dementias: dementia with Lewy bodies (DLB) and Parkinson’s disease dementia (PDD). To understand how α-syn misfolding leads to cellular pathologies culminating in cell death, Susan Lindquist pioneered a yeast model of α-syn (Outeiro and Lindquist, 2003). α-Syn, a protein exclusive to mammals without direct homologs in lower organisms, sparked skepticism when introduced as a yeast model of α-syn pathobiology. Yet, this model harnessed a fundamental and evolutionarily conserved biological process of all living systems: protein folding (Khurana and Lindquist, 2010). Moreover, leveraging the yeast’s high-throughput capabilities as a model system, Susan Lindquist’s group was among the first to attribute a role to α-syn within the secretory pathway (Auluck et al., 2010). Overexpressing SNARE proteins involved in endoplasmic reticulum (ER) to Golgi transport overcome the vesicular arrest between the ER and Golgi caused by α-syn (Cooper et al., 2006; Gitler et al., 2008). Moreover, using this model, we and others found that α-syn leads to a pathological increase in cytosolic Ca^2+^, resulting in cell death (Chan et al., 2007; Danzer et al., 2007; Dufty et al., 2007; Guzman et al., 2010; Surmeier et al., 2010; Goldberg et al., 2012; Martin et al., 2012; Buttner et al., 2013; Hurley et al., 2013; Yuan et al., 2013; Caraveo et al., 2014; Surmeier et al., 2016; Caraveo et al., 2017; Surmeier et al., 2017; Betzer et al., 2018; Shum et al., 2023). Some data suggest that the source of this pathological elevation could be either Ca^2+^ uptake at the plasma membrane via the L-type voltage-gated Ca^2+^ channel Cav1.3 (Brown et al., 2006; Chan et al., 2007; Ilijic et al., 2011; Hurley and Dexter, 2012; Van Maele-Fabry et al., 2012; Dryanovski et al., 2013; Cali et al., 2014; Goldman, 2014; Schapira, 2015; Ortner and Striessnig, 2016; Surmeier et al., 2016; Zamponi, 2016) or α-syn itself, as its oligomeric conformation has the ability to form pores at the plasma membrane (Danzer et al., 2007; Angelova et al., 2016; Di Scala et al., 2016). However, findings from our group and others implicate defects in intracellular Ca^2+^ stores (Cali et al., 2012; Caraveo et al., 2014; Paillusson et al., 2017). Whether the rise in cytosolic Ca^2+^ is due to a secondary effect stemming from ER–Golgi transport arrest, a direct effect of α-syn gating Ca^2+^ channels, or both remains to be determined.

Irrespective of whether the cytosolic Ca^2+^ increase originates from the extracellular environment or from intracellular stores, all studies converge on one point: α-syn triggers a pathological elevation in cytosolic Ca^2+^. An essential transducer of Ca^2+^ gradients into cellular responses is the highly evolutionarily conserved Ca^2+^–calmodulin-dependent serine/threonine phosphatase, calcineurin (Aramburu et al., 2004). Our group and others have established a central role of calcineurin activity in α-syn pathobiology (Dufty et al., 2007; Guzman et al., 2010; Surmeier et al., 2010; Goldberg et al., 2012; Martin et al., 2012; Hurley et al., 2013; Caraveo et al., 2014; Surmeier et al., 2016; Burbulla et al., 2017). Using a range of model organisms, starting from yeasts and worms to primary cortical and dopaminergic neurons as well as *in vivo* rodent models of α-syn pathobiology, we found that disease-associated forms of α-syn lead to a pathological increase in cytosolic Ca^2+^ and heightened calcineurin activity, culminating in neuronal death (Guzman et al., 2010; Surmeier et al., 2010; Goldberg et al., 2012; Hurley et al., 2013; Caraveo et al., 2014; Surmeier et al., 2016). However, reducing calcineurin activation through pharmacological means using sub-saturating doses of the Food Drug Administration (FDA)-approved calcineurin inhibitor FK506 (also known as tacrolimus) rescued neurons from α-syn toxic effects. Conversely, complete inhibition of calcineurin with saturating doses of FK506 eliminates calcineurin activity, also resulting in cell death (Guzman et al., 2010; Surmeier et al., 2010; Goldberg et al., 2012; Hurley et al., 2013; Caraveo et al., 2014; Surmeier et al., 2016). While several studies support the neuroprotective role of inhibiting calcineurin in both *in vitro* and *in vivo* models of PD with FK506 (Costantini et al., 1998; Guo et al., 2001a; Guo et al., 2001b; Singh et al., 2003; Wright et al., 2008; Overk et al., 2015; Van der Perren et al., 2015), our study provided the mechanistic understanding for the efficacy of sub-saturating doses of FK506 *in vivo*. Specifically, we found that the 12-kDa cis–trans proline isomerase FK506-binding protein (FKBP12) can endogenously regulate calcineurin activity (Dufty et al., 2007). Given that FK506 targets the interface between calcineurin and FKBP12, thereby inhibiting the enzymatic activities of both enzymes (Liu et al., 1991; Guasch et al., 2015), at sub-saturating doses, FK506 can enable substrate dephosphorylation of high-affinity substrates.

FK506 is already widely used in the clinic at saturating doses to suppress organ rejection in transplant patients, a process in which calcineurin also plays a critical role (Staatz and Tett, 2004). However, the standard FK506 dosing to suppress the immune system would have adverse effects and likely impair calcineurin activity, hampering its ability to activate neuroprotective responses. To monitor the desired neuroprotective effects of FK506 treatment, a measurable indicator or biomarker of calcineurin activity is essential.

In this study, we show that two calcineurin-derived substrates, anti-inflammatory insulin-like growth factor 1 (IGF-1) and pro-inflammatory cytokine interleukin 2 (IL-2), are responsive to FK506 treatment in both the cerebral cortex and serum in a mouse model of α-synuclein pathology. Specifically, we found that inhibiting calcineurin with FK506 reduced IL-2 levels and increased IGF-1 levels in an α-syn transgenic mouse model, both in the cerebral cortex and serum. Importantly, IL-2 and IGF-1 levels have been shown to be altered in PD patients (Kim et al., 2018; King et al., 2019; Shi et al., 2022). Moreover, FK506 is an approved clinical drug and can penetrate the brain. A clinical trial using IGF-1 and IL-2 as biomarkers for FK506 dosing would encounter no significant logistical or regulatory obstacles. Given the extensive clinical experience with FK506 for other conditions, this objective should be readily attainable for patients afflicted with DLB and PDD.

## Results

To identify the substrates dephosphorylated by calcineurin associated with toxic vs. protective responses, we previously adopted both unbiased and candidate-based approaches (Caraveo et al., 2014; Caraveo et al., 2017). In the candidate approach, we focused on the transcription factor of activated T cells (NFAT), an extensively studied calcineurin substrate (Rao et al., 1997). Originally discovered in immune cells, NFAT plays critical roles in many other cell types, including brain cells, both during development and adulthood (Rao et al., 1997). When dephosphorylated by calcineurin, NFAT translocates to the nucleus, where it activates the transcription of signaling proteins responsible for coordinating cell communication within the immune system and other physiological processes (Rao et al., 1997). Initially identified in the yeast model of α-syn, we found that the activation of NFAT by calcineurin is a key driver for α-syn toxicity (Caraveo et al., 2014). Overexpressing the yeast equivalent of NFAT exacerbated α-syn toxicity, whereas its deletion protected yeast cells against the toxic effects of α-syn (Caraveo et al., 2014). Importantly, we found hallmarks of NFAT activation in neurons and glial cells in the cerebral cortex of α-synuclein transgenic mice as well as in the postmortem brains of individuals diagnosed with PDD and DLB (Caraveo et al., 2014).

**FIGURE 1:**
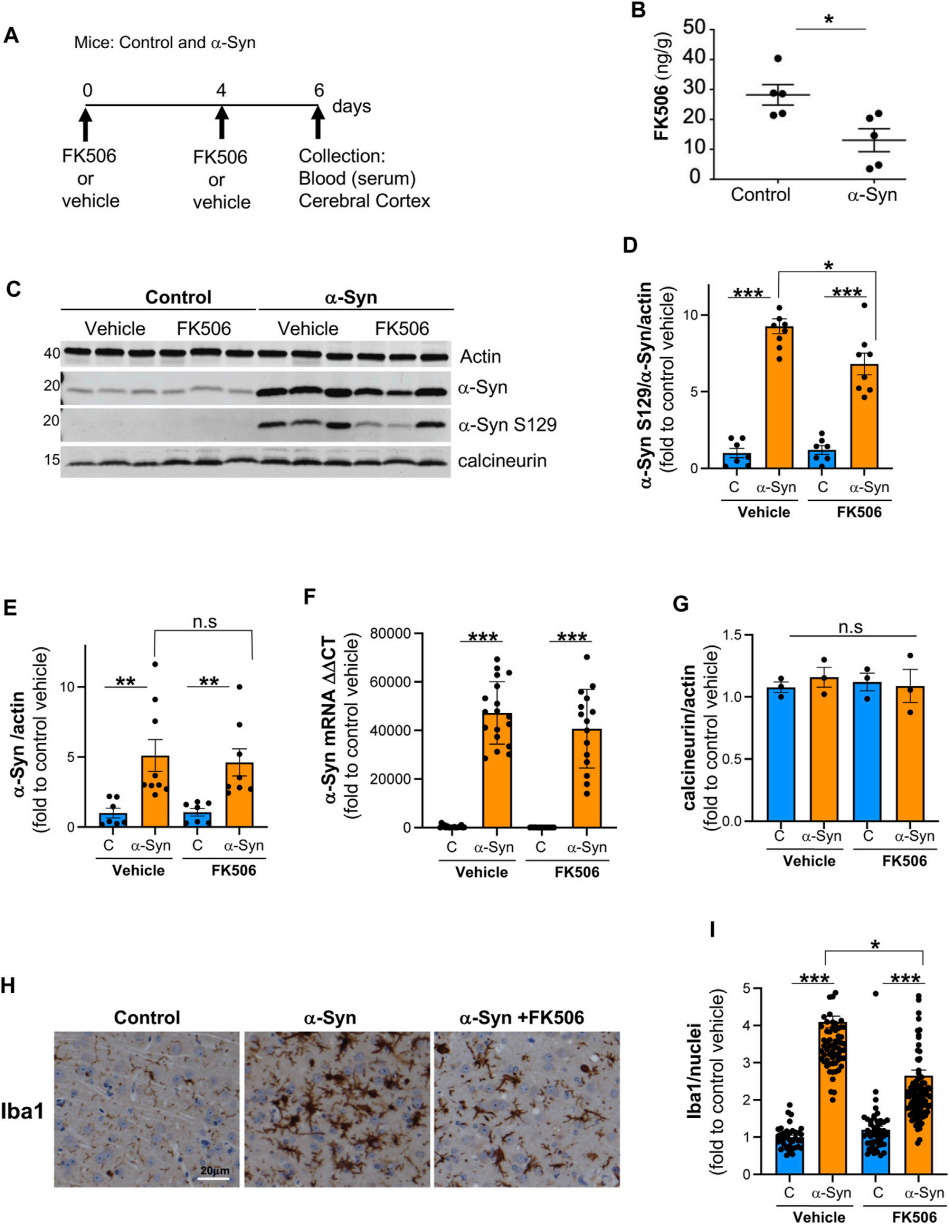
FK506 is a brain-penetrant drug and confers protection against α-syn toxicity. **(A)** Schematic representation of the experimental design; CaMKII–Cre (control) and CaMKII–Cre–α-syn (α-syn) animals were subcutaneously injected twice with FK506 (4 mg/kg) or vehicle (dimethyl sulfoxide or DMSO) 4 days apart and killed on day 6. **(B)** Brain FK506 levels determined via mass spectrometry from transgenic animals in **(A)**. N ≥ 5; *p < 0.05; one-way ANOVA with Tukey’s *post hoc* test. **(C)** Representative Western blot for α-syn, phosphoserine-129 α-syn, and calcineurin from the cerebral cortex lysates of animals in **(A)**; actin serves as the loading control. **(D)** Densitometry quantitation from WB in **(C)** of p-S129 α-syn/α-syn/actin. Data are normalized to control mice treated with vehicle (DMSO). N ≥ 5; *p < 0.05 and ***p < 0.001; one-way ANOVA with Tukey’s *post hoc* test. **(E)** Densitometry quantitation from WB in **(C)** for α-syn/actin (L) normalized to control mice treated with vehicle. N ≥ 5; n.s., non-significant; one-way ANOVA with Tukey’s *post hoc* test. **(F)** qPCR for α-syn from the indicated mice cortices from **(A)**. N ≥ 5; ***p < 0.001; one-way ANOVA with Tukey’s *post hoc* test. **(G)** Densitometry quantitation from WB in **(C)** for calcineurin/actin (L) normalized to control mice treated with vehicle. N ≥ 5; n.s., non-significant; one-way ANOVA with Tukey’s *post hoc* test. **(H)** Representative immunohistochemistry for Iba1 (marker for microglia) of matched sections from the cerebral cortex of animals in **(A)**. Scale bar is 20 µm. **(I)** Quantification of Iba1 fluorescence intensity/Iba1-positive nuclei from animals in **(A)**. N ≥ 5 animals and 5 sections/animal. *p < 0.05 and ***p < 0.001; one-way ANOVA with Tukey’s *post hoc* test.

**FIGURE 2:**
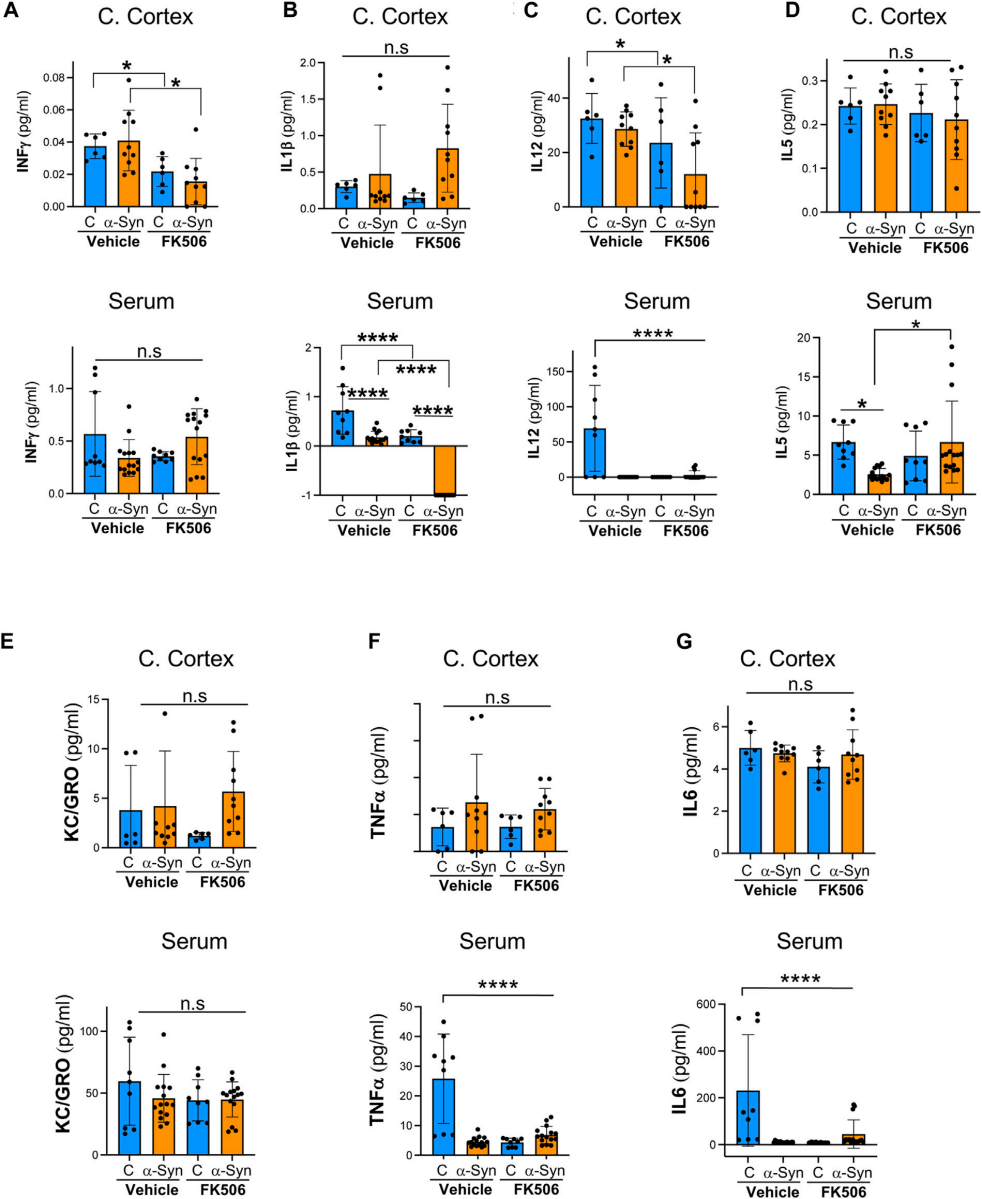
Pro-inflammatory profile of α-syn transgenic mice after FK506 dosing. **(A–G)** Pro-inflammatory cytokine levels measured using the MSD multiplex system from the cerebral cortices and sera of CaMKII–Cre (control) and CaMKII–Cre–α-syn (α-syn) animals subcutaneously injected twice with FK506 (4 mg/kg) or vehicle (DMSO) 4 days apart and killed on day 6. N ≥ 5; n.s., non-significant, *p < 0.05 and ****p < 0.0001; one-way ANOVA with Tukey’s *post hoc* test.

NFAT activation has been implicated in modulating inflammation, oxidative stress, and neuronal survival in other neurological diseases (Fernandez et al., 2007; Sompol and Norris, 2018). Chronic NFAT activation leads to an increase in pro-inflammatory cytokines, creating a pathological loop resulting in chronic neuroinflammation and neuronal death (Sompol and Norris, 2018). To determine whether pro-inflammatory cytokines driven by NFAT/calcineurin activation are a hallmark of α-syn pathobiology and responsive to FK506 treatment, we employed a well-established mouse model of α-syn pathobiology. This synucleinopathy model harnesses the Ca^2+^/calmodulin-dependent kinase II (CaMKII)–tTA promoter to drive human α-syn A53T (Lin et al., 2009), leading to high expression levels in the cerebral cortex, a region significantly affected in DLB and PD (Alafuzoff and Hartikainen, 2017). Reflecting the age-dependent onset of pathology as in human disease, we treated control and α-syn-expressing mice (12–18 months old) with vehicle or two single doses of FK506, administered 4 days apart (Figures 1A, B). The selected dose corresponds to standard saturating inhibitory calcineurin doses (Jain et al., 1993). In the cerebral cortex, FK506 treatment showed two signs of neuroprotection: 1) reduction in phosphorylated α-syn S129, the post-translational modified form associated with pathology (Fujiwara et al., 2002), without alternating the expression of α-syn or calcineurin (Figures 1C–G), and 2) reduction in Iba1 expression (Figures 1H, I), a microglial marker whose increased expression is a hallmark of neuroinflammation due to neuronal stress/damage (Schwabenland et al., 2021).

Once we confirmed FK506 brain penetration and neuroprotection, we conducted a multiplex cytokine assay using a Meso Scale Discovery (MSD) QuickPlex SQ 120 on cerebral cortex samples and serum from these mice. This electrochemiluminescence-based assay allows the simultaneous measurement of cytokines in a single sample. We analyzed a total of 10 pro- and anti-inflammatory cytokines. We seek the cytokine(s) that was differentially expressed between control and α-syn animals and responsive to FK506 treatment in both the brain and periphery.

**FIGURE 3:**
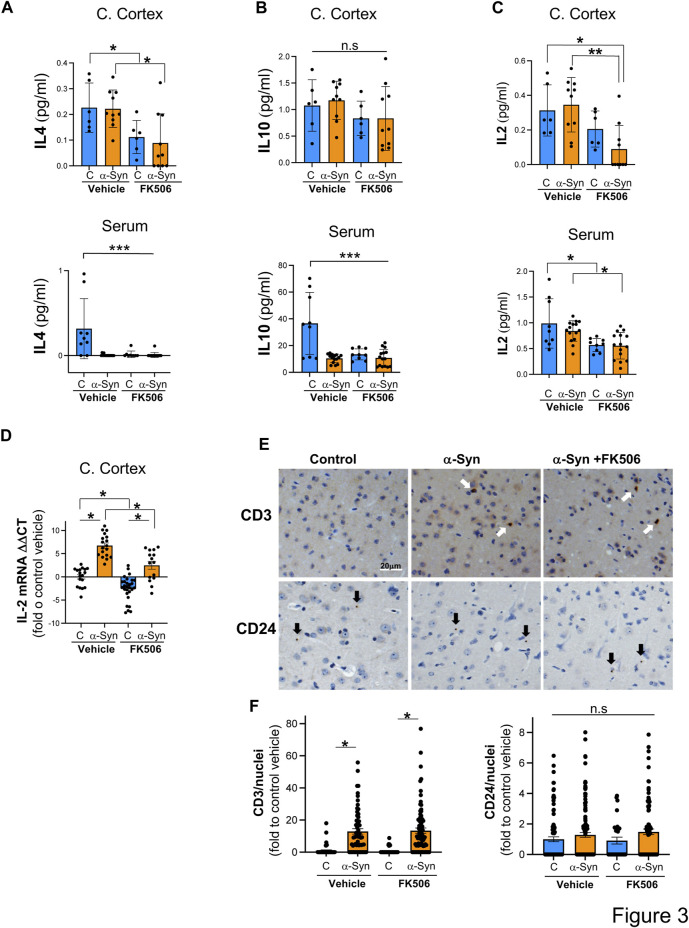
IL-2 levels are increased in the cerebral cortex of α-syn mice, and FK506 treatment reduces it. **(A–C)** Pro-inflammatory cytokine levels measured using the MSD multiplex system from cerebral cortices and sera of CaMKII–Cre (control) and CaMKII–Cre–α-syn (α-syn) animals subcutaneously injected twice with FK506 (4 mg/kg) or vehicle (DMSO) 4 days apart and killed on day 6. N ≥ 5; n. s., non-significant; *p < 0.05 and ****p < 0.0001; one-way ANOVA with Tukey’s *post hoc* test. **(D)** qPCR for IL-2 normalized to control/DMSO from the cerebral cortex from mice in **(A)**. N ≥ 5. *p < 0.05; one-way ANOVA with Tukey’s *post hoc* test. **(E)** Representative immunohistochemistry for CD3 (marker for T cells) and for CD24 (marker for B cells) of matched sections from the cerebral cortex of animals in **(A)**. Scale bar is 20 µm. **(F)** Quantification of CD3 and CD24 fluorescence intensity/positive nuclei from animals in **(A)**. N ≥ 5 animals and five sections/animal. n.s., non-significant; *p < 0.05; one-way ANOVA with Tukey’s *post hoc* test.

Control and α-syn animals had a very different cytokine response in the brain versus the periphery. In the cerebral cortex, none of the sampled cytokines showed a statistically significant difference between controls and α-syn transgenic mice (Figures 2, 3). In sharp contrast, most serum cytokines, with the exception of KC/GRO, IFN-γ, and IL-2, which remained unchanged (Figures 2A, E; Figure 3C), were downregulated in α-syn transgenic mice compared to controls (Figures 2B–D, F, G; Figures 3A, B). Therefore, no cytokine showed a consistent response in the brain and periphery between controls and α-syn transgenic mice.

In the cerebral cortex of α-syn transgenic mice, treatment with FK506 affected the expression of only four cytokines: INF-γ, IL-12, IL-4, and IL-2 (Figures 2A, C; Figures 3A, C). However, in the serum of α-syn transgenic mice, treatment with FK506 affected the expression of most cytokines except for INF-γ and KC/GRO (Figures 2A, E). Out of the three cytokines that consistently changed their expression in response to FK506 treatment in both the brain and periphery, namely, IL-12, IL-4 and IL-2, only IL-2 was different between α-syn transgenic mice treated with vehicle and α-syn transgenic mice treated with FK506 (Figure 2C; Figures 3A, C). While IL-2 levels were not statistically different between control and α-syn transgenic mice (Figure 3C), we noticed two sub-groups within the α-syn transgenic mice: high and low levels of IL-2 protein in the cerebral cortex (Figure 3C). To investigate whether this difference was real, we performed a more sensitive assay for mRNA using quantitative real-time polymerase chain reaction (qPCR) for IL-2 from the cerebral cortex of these mice. Indeed, mRNA IL-2 levels were significantly increased in α-syn mice compared to controls, and treatment with FK506 reduced them (Figure 3D). In summary, among the cytokines in the MSD panel, IL-2 was the only cytokine sensitive to FK506 treatment in both the cerebral cortex and serum of α-syn transgenic mice, and its mRNA expression was increased in α-syn transgenic mice compared to controls.

Cytokines serve as key messengers through which immune cells communicate to drive immune and inflammatory responses. To investigate whether the immune infiltration was present in the α-syn cerebral cortex, we immunoassayed for CD3, a marker of T cells, and CD24, a marker for B cells. While we detected very few B cells in the cerebral cortex of these mice, neither the presence of α-syn nor FK506 treatment had an effect on the number of infiltrated B cells (Figures 3E, F). In contrast, we found a significant increase in T cells in α-syn transgenic mice compared to controls (Figures 3E, F). However, FK506 treatment had no impact on T-cell infiltration in α-syn transgenic mice (Figures 3E, F). Whether IL-2 levels in the brain originate from the infiltrated T cells, from brain resident cells (either neurons and/or glial cells), or both remains to be determined.

**FIGURE 4:**
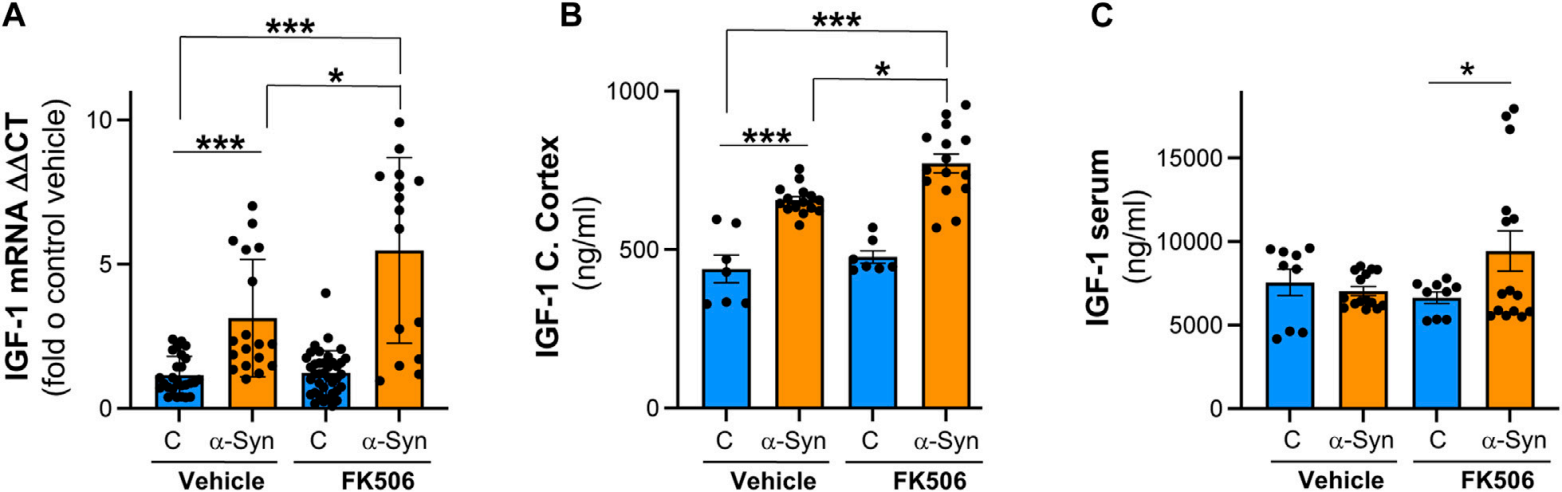
IGF-1 levels are increased in the cerebral cortex of α-syn mice, and FK506 treatment increases it. **(A)** qPCR for IGF-1 normalized to control/DMSO from the cerebral cortex of CaMKII–Cre (control) and CaMKII–Cre–α-syn (α-syn) animals subcutaneously injected twice with FK506 (4 mg/kg) or vehicle (DMSO) 4 days apart and sacrificed on day 6; N ≥ 5 animals, duplicates. *p < 0.05 and ***p < 0.001; one-way ANOVA with Tukey’s *post hoc* test. **(B)** IGF-1 protein by ELISA from the cerebral cortex of animals in **(A)**. N ≥ 5 animals, duplicates. *p < 0.05 and ***p < 0.001; one-way ANOVA with Tukey’s *post hoc* test. **(C)** IGF-1 protein by ELISA from the serum of animals in **(A)**. N ≥ 5; two technical replicates. *p < 0.05; one-way ANOVA with Tukey’s *post hoc* test.

In an unbiased transcriptional approach using RNA sequencing (RNA-Seq), we previously found that one of the most prominent upregulated genes in α-syn neuronal cultures treated with neuroprotective doses of FK506 was the IGF-1 signaling pathway (Shum et al., 2023). IGF-1 signaling has been shown to be neuroprotective, partially due to its role in inhibiting neuroinflammation (Sukhanov et al., 2007; Park et al., 2011). Additionally, decreased levels of serum IGF-1 have been linked to poor cognitive prognosis for PD patients (Ma et al., 2015). To validate these RNA-Seq-based findings, we performed qPCR and ELISA for IGF-1 in the cerebral cortex of α-syn transgenic animals. α-Syn caused a modest but significant transcriptional increase in IGF-1 levels, accompanied by a concordant increase in IGF-1 protein levels in α-syn transgenic animals (Figures 4A, B). Notably, FK506 treatment in α-syn transgenic animals further elevated mRNA and protein IGF-1 levels (Figures 4A, B). To investigate whether the upregulation of IGF-1 due to FK506 treatment also occurred in the periphery, we conducted ELISA in the serum of these animals. While we did not detect differences between control and α-syn transgenic animals in IGF-1 levels, FK506 treatment increased IGF-1 levels only in α-syn transgenic animals (Figure 4C). Collectively, these findings demonstrate that FK506 treatment increases IGF-1 levels in both the cerebral cortex and periphery exclusively in α-syn transgenic animals.

## Discussion

Based on the previous work through candidate and unbiased approaches (Caraveo et al., 2014; Shum et al., 2023), we have now identified two plausible biomarkers responsive to the neuroprotective properties of FK506 *in vivo*: IL-2 and IGF-1. IL-2 expression is primarily regulated by NFAT, a *bona fide* calcineurin substrate (Serfling et al., 2000; Bueno et al., 2002; Hogan et al., 2003; Macian, 2005).

We found that mRNA IL-2 levels are increased in the cerebral cortex of α-syn transgenic mice, and FK506 treatment reduced them. Although we did not find a statistically significant increase in IL-2 protein levels in the cerebral cortex of α-syn transgenic mice relative to controls, FK506 treatment led to a reduction in IL-2 levels. The lack of correlation between mRNA IL-2 levels and intracellular protein concentration in the cerebral cortex could be attributed to two factors: 1) the half-life of the IL-2 protein and 2) the intrinsic biological variability of the IL-2 response (supported by the appearance of high- and low-IL-2 expressing subgroups within the α-syn condition). Like the cerebral cortex, we did not find a difference in serum IL-2 levels between control and α-syn transgenic mice, yet FK506 treatment reduced IL-2 levels in both control and α-syn transgenic mice. Although we cannot argue that the lack of difference in IL-2 levels between control and α-syn transgenic mice is likely due to the sensitivity of the assay (we could not measure mRNA from serum due to insufficient material), the lack of differences may reflect the following: a limitation of the model we used is that α-syn overexpression is restricted to CaMKII-positive regions of the brain (Lin et al., 2009). Alternatively, it is possible that IL-2 production in response to α-syn in the cerebral cortex is restricted to the brain.

Cytokines serve as key messengers through which immune cells communicate to drive immune and inflammatory responses; however, they can be produced locally in the brain to modulate brain physiology (Bourgognon and Cavanagh, 2020). IL-2, for example, has been shown to improve cognitive performance in rodents (Hanisch and Quirion, 1995; Dansokho et al., 2016) and provide trophic support to neurons and glial cells (Bourgognon and Cavanagh, 2020). IL-2-knockout mice exhibit cytoarchitectural changes in the hippocampal dentate gyrus and have impaired learning and memory (Williams et al., 2021). Despite strong evidence supporting IL-2’s local brain role, IL-2 can also originate from infiltrating immune cells or immune cells within brain barriers like meninges. Interestingly, T-cell infiltration in the central nervous system (CNS) has been shown to occur in response to α-syn-mediated neuroinflammation and in PD (Galiano-Landeira et al., 2020; Williams et al., 2021; Karikari et al., 2022). While FK506 treatment did not mitigate T-cell infiltration in the cerebral cortex of α-syn transgenic mice, it can still decrease T-cell activation by reducing IL-2 production rather than recruitment. Alternatively, both immune and CNS local IL-2 production might contribute to the overall increase in cerebral cortex IL-2 production in α-syn mice. If IL-2 has been shown to play a neuroprotective role in the brain, why would increased levels be associated with α-syn toxicity? It has been shown that a single cytokine can have seemingly opposite effects on neuronal activity depending on its concentration (Prieto and Cotman, 2017). Importantly, PD patients have elevated IL-2 levels in their CSF and serum (Kim et al., 2018; King et al., 2019; Shi et al., 2023), implying that IL-2 levels might be too high to provide neuroprotection. Reducing IL-2 levels with FK506 could redirect its neuroprotective properties. While future work is needed to uncover IL-2’s effect on cerebral cortex dysfunction, IL-2 can serve as a biomarker for calcineurin activity to guide FK506 dosing in both the periphery and brain.

Beyond IL-2, we found that IGF levels are responsive to FK506. Specifically, we found increased IGF-1 levels in the cerebral cortex of α-syn mice compared to controls. Treatment with FK506 further elevated IGF-1 levels both in the cerebral cortex and serum of α-syn mice. The increased peripheral IGF-1 levels in α-syn transgenic mice treated with FK506 suggest a brain origin, as α-syn is not overexpressed in the periphery. IGF-1 expression can be regulated by calcineurin via NFAT (Alfieri et al., 2007) or by NF-κB via IkB kinase, a substrate for calcineurin (Mitsiades et al., 2002). Although IGF-1 can be produced by various cell types in the body, in CNS, neurons and glial cells, mainly astrocytes, serve as the main sources (Labandeira-Garcia et al., 2017; Shum et al., 2023). Importantly, treatment with IGF-1 has been shown to ameliorate α-syn proteotoxicity (Kao, 2009) and dopamine-induced neurotoxicity (Offen et al., 2001). Some of the neuroprotective effects of IGF-1 lie in its ability to promote dendritic growth, axonal sprouting, and synapse formation/strengthening (Lewitt and Boyd, 2019). While these neuroprotective effects are also well documented for other neurodegenerative and neurotraumatic conditions, including amyotrophic lateral sclerosis and ischemia (Bassil et al., 2014; Bernhard et al., 2016; Rodriguez-Perez et al., 2016; Bianchi et al., 2017; Labandeira-Garcia et al., 2017), reductions in IGF-1-mediated signaling, on the other hand, have been shown to delay amyloid-β proteotoxicity in mice (Cohen et al., 2009; Gontier et al., 2015). What underlies the opposite effects of IGF-1 in different neurodegenerative diseases might have to do with the pathological roots of these diseases. While we did not directly test the effect of IGF-1 treatment against α-syn pathobiology in our model, we can safely say that the increase in IGF-1 levels in response to FK506 is associated with signs of neuroprotection in mice. Importantly, decreased serum IGF-1 levels are associated with poor cognitive prognosis in PD patients (Ma et al., 2015). Moreover, IGF-1 levels are altered in PD and correlate with disease progression (Suzuki et al., 2019; Shi et al., 2022). Therefore, IGF-1 can serve as a valuable biomarker to monitor the neuroprotective effects of FK506 dosing in human patients.

Although the goal of this work was to uncover biomarkers for calcineurin activity that respond to FK506 dosing, it is important to mention that, while not monitored here, our own previous work and others have shown that FK506 neuroprotective effects against α-syn can extend beyond calcineurin via inhibition of FKBP12 and possibly other FKBPs. Inhibition of FKBP12 by FK506 can directly affect α-syn pathobiology by preventing its aggregation (Gerard et al., 2006; Gerard et al., 2008) and indirectly by affecting other relevant pathways to α-syn pathobiology (Caraveo et al., 2017). Furthermore, the inhibition of other immunophilins has also been implicated in neuroprotection in PD models (Steiner et al., 1997; Costantini et al., 1998; Tanaka and Ogawa, 2004; Gerard et al., 2010). Therefore, our studies support the use of sub-saturating doses of FK506, given its ability to target two essential pathways in α-syn pathobiology: calcineurin-dependent and calcineurin-independent pathways. Moreover, while saturating doses of FK506 are widely used in the clinic to suppress the rejection of organs in transplant patients, a process in which calcineurin also plays a critical role, the sub-saturating doses we propose would cause minimal and transient immunosuppression since the drug’s half-life in circulation is 12 h (Venkataramanan et al., 1995).

In conclusion, based on previous studies, insights from studies utilizing Susan Lindquist’s yeast model of α-syn pathobiology revealed an underappreciated property of Ca^2+^-dependent calcineurin activity: the extent of its activation determines substrate usage (Caraveo et al., 2014; Caraveo et al., 2017). The highly conserved Ca^2+^-calcineurin signaling pathway is disrupted in α-syn-dependent human disease (Caraveo et al., 2014). Moreover, our study provides two biomarkers that are measurable throughout life to track neuroprotective FK506 dosing in human patients in both serum and the brain: a decrease in IL-2 and an increase in IGF-1. Therefore, a clinical trial of FK506 treatment in early-stage PD patients would pose no significant logistical or regulatory challenges. The established approval of FK506 for other uses, its high brain penetrance (Murakami et al., 2004; Taglialatela et al., 2015; Radhakrishnan et al., 2021), and its safety profile provide a strong rationale for repurposing it against synucleinopathies.

## Materials and methods

### Antibodies

For immunofluorescence, the following primary antibodies were used: MAP2 (Millipore, AB5622), Iba1 (Waco, 019-19741, 1:250), CD3 (Abcam, ab16669, 1:250), and CD24 (Santa Cruz, #sc-11406, 1:100). The secondary antibodies used were Alexa 488 (Invitrogen, a21202) and Alexa 594 (Invitrogen, a21442). For Western blot analysis, actin (Abcam, ab6276), α-synclein (BD, 610787), phospho-α-synclein S129 (a kind gift from Dr. Takeshi Iwatsubo, The University of Tokyo, Japan), and calcineurin B (CNB, Abcam, ab154650) were used.

### Mice

All protocols were approved by the Massachusetts Institute of Technology’s University Administrative Panel on Laboratory Animal Care. C57BL6 mice with human α-syn A53T driven by the calcium/calmodulin-dependent kinase II (CaMKII)–tTA promoter were obtained from the Jackson Laboratory (Tg(tetO-SNCA*A53T)E2Cai/J, #012442) donated by Huaibin Cai’s laboratory. Animals aged 12–18 months received two doses of FK506 (4 mg/kg) with a 4-day interval between injections, and mice were sacrificed on day 6. Blood (red blood cells removed through low-speed, ∼1,000 rpm centrifugation) and brains were collected. Half brains were immediately flash-frozen, and second halves were fixed in 4% (vol/vol) formalin. All mouse brains were analyzed for α-syn levels. Only animals with high and matched α-syn levels were selected for further analysis. At the end, six controls (CAMKII^+^/αSyn^−^) injected with DMSO or FK506, six α-syn (CAMKII^+^/αSyn^+^) injected with DMSO, and five α-syn (CAMKII^+^/αSyn^+^) injected with FK506 were chosen from three independent experiments that had at least five animals in each group. FK506 brain content was determined in the mouse cerebellum using liquid chromatography-mass spectrometry (LC-MS) by Sanford Burnham Prebys (SBP, La Jolla, CA).

### SDS-PAGE/Western blotting

Infected neuronal cultures (5 weeks and 2 days post-transduction) and mouse cortexes (∼0.25 mg of tissue) were lysed using a radioimmunoprecipitation assay (RIPA) buffer (50 mM Tris/HCl, pH 7.6; 150 mM NaCl; 20 mM KCl; 1.5 mM MgCl_2_; 1% NP40; and 0.1% SDS). In all experiments, the lysis buffer was supplemented with the Halt protease and phosphatase inhibiter cocktail (Thermo Fisher Scientific; 78441). Samples were incubated on ice for 30 min and pushed through a 27G needle (10 times) to ensure full lysis. Samples were then centrifuged at maximum RPM (∼20,000 × g) for 20 min, and the subsequent supernatants were used for Western blot analysis. The protein concentration was analyzed using the Pierce BCA Protein Assay Kit (Thermo Fisher Scientific) and the Fisherbrand™ accuSkan™ GO UV/Vis Microplate Spectrophotometer (Fisher Scientific). After the addition of the appropriate amount of the 6X Laemmli sample buffer (Bioland Scientific LLC, sab03-02) with 5% ß-mercaptoethanol (Sigma), protein samples (10–30 µg) were boiled and separated on pre-casted 4%–20% Criterion TGX Stain-Free Gels (Bio-Rad) and transferred to a nitrocellulose membrane (Amersham Protran 0.2 μm NC, #10600001). Membranes were blocked with 5% non-fat milk in 1X Tris-buffered saline (TBS) (50 mM Tris, pH 7.4; 150 mM NaCl) for 1 h at room temperature. Membranes were subsequently immunoblotted overnight with a primary antibody at 4°C with continuous shaking. The following day, membranes were washed three times with 1X TBST (TBS with 0.1% Tween) for 5 min and incubated in secondary IRDye antibody for 1 h shaking at room temperature. Membranes were washed three times with 1X TBST before imaging using the LI-COR Odyssey^®^ CLx Imaging System. Images were processed using IMAGE Studio software (LI-COR Biosciences), and signal densities were quantified using Fiji (Schindelin et al., 2012).

### ELISA and MSD array

Cleared and normalized (to total protein) mouse cortex lysate and blood samples were used to determine levels of IGF-1 using the Mouse IGF-1 PicoKine ELISA Kit (Boster Biological Technology, EK0378) and the Fisherbrand™ accuSkan™ GO UV/Vis Microplate Spectrophotometer (Fisher Scientific). Levels of IL-2, INF-γ, IL-1β, IL-4, IL-5, IL-6, KC/GRO, IL-10, IL-12p70, and TNF-α were determined using the V-PLEX Proinflammatory Panel 1 (mouse) Kit (MSD, K15048G), according to the manufacturer’s protocols, and the Meso Scale Discovery QuickPlex SQ 120 instrument (MSD).

### mRNA isolation, cDNA synthesis, and qPCR

Total RNA was isolated from mouse cortices (∼0.25 mg per sample) using RNeasy kits (QIAGEN, 73304 and 73404) according to the manufacturer’s specifications. cDNA was synthesized using the High-Capacity cDNA RT Kit (Thermo Fisher Scientific, 4368814) from 0.5 µg of RNA according to the manufacturer’s specifications. qPCR was performed using the iTaq Universal SYBR^®^ Green Master Mix (Bio-Rad, 1725121) on a LightCycler 480 II (Roche). Ten pmol of primer mixes (see Table 1) and 10 ng of cDNA were used to amplify cDNA fragments. Results were expressed as ΔΔCp (fit point method, Roche) and obtained using the comparative C_T_ method, also referred to as the 2^−ΔΔCT^ method (normalized to HPRT values).

**TABLE 1:** qPCR primers.

Primer name	Sequence (5'-> 3′)
Mouse	
HPRT F	GGG​GCT​GTA​CTG​CTT​AAC​CAG
HPRT Rev	TCA​GTC​AAC​GGG​GGA​CAT​AAA
IGF-1 F	CAC​ATC​ATG​TCG​TCT​TCA​CAC​C
IGF-1 Rev	GGA​AGC​AAC​ACT​CAT​CCA​CAA​TG
SNCA F	GGG​ACT​AGT​ACC​ATG​GAT​GTA​TTC​ATG​AAA​GGA​CTT​TCA​AAG​GCC​AAG​GAG​GG
SNCA Rev	CTC​CTT​CTT​CAT​TCT​TGC​CCA​A
IL-2 F	TGA​GCA​GGA​TGG​AGA​ATT​ACA​GG
IL-2 Rev	GTC​CAA​GTT​CAT​CTT​CTA​GGC​AC

### Immunohistochemistry and immunofluorescence

Immunohistochemistry (IHC): Histology services were provided by the Northwestern University Mouse Histology and Phenotyping Laboratory, which is supported by NCI P30-CA060553 awarded to the Robert H Lurie Comprehensive Cancer Center. Fixed half brains were processed into paraffin-embedded blocks and cut, and matched slices from the whole group of mice were processed for IHC staining. Initial evaluation of antibodies was tested on a separate group of mice that expressed low levels of α-syn. All mounted slides were deparaffinized and rehydrated using a Leica Autostainer XL (Leica). Antigen retrieval was done in a sodium citrate buffer (0.1 M, pH 6.0) for 5 min (GFAP) or 10 min (Iba1) at 95°C using a Decloaking Chamber™ NxGen (Biocare). Alternately, antigen retrieval was done for 20 min (CD3) and 5 min (CD24) at 110°C. The following primary antibodies were used: Iba1 (Wako, 019-19741, 1:250), CD3 (Abcam, ab16669, 1:250), and CD24 (Santa Cruz, #sc-11406, 1:100). The secondary antibody used was Biotin-SP (long spacer) AffiniPure Donkey Anti-Rabbit IgG (H + L) (Jackson immuno #711-065–152). The IHC detection method employed the standard avidin–biotin complex (ABC) and DAB (3,3′-diaminobenzidine) HRP substrate protocol. Images were acquired using the Olympus BX41 Dual Head Microscope equipped with the X-cite 120LED camera and operated with cellSens imaging software (version 1.12) provided by the core. Images were analyzed using the IHC Image Analysis Toolbox (FIGI) (Schindelin et al., 2012). CD3- and CD24-positive cells were counted manually. Total Iba1 signals were calculated using the threshold method and expressed as a normalized percentage of area. The resulting data were processed using Microsoft Excel and Prism GraphPad (http://www.graphpad.com).

### Statistical analysis

One-way ANOVA with Tukey’s *post hoc* test was used for three or more dataset quantifications. Statistical calculations were performed using GraphPad Prism 7 Software (http://www.graphpad.com), and *p*-values < 0.05 were considered significant. Results are expressed as the average + the standard error mean (SEM). Regression analysis was done using the Microsoft Excel Statistics add-in package. A minimum of two independent biological replicates were used for each experiment, with at least six replicates per sample within each experiment. The specific number of biological replicates for each experiment is listed in the figure legends.

## Data Availability

The original contributions presented in the study are included in the article/Supplementary Material; further inquiries can be directed to the corresponding author.
